# Molecular Phylogeny Restores the Supra-Generic Subdivision of Homoscleromorph Sponges (Porifera, Homoscleromorpha)

**DOI:** 10.1371/journal.pone.0014290

**Published:** 2010-12-14

**Authors:** Eve Gazave, Pascal Lapébie, Emmanuelle Renard, Jean Vacelet, Caroline Rocher, Alexander V. Ereskovsky, Dennis V. Lavrov, Carole Borchiellini

**Affiliations:** 1 Aix-Marseille Université, Centre d'Océanologie de Marseille, Station marine d'Endoume - CNRS UMR 6540-DIMAR, Marseilles, France; 2 Department of Embryology, Faculty of Biology and Soils, Saint-Petersburg State University, St Petersburg, Russia; 3 Department of Ecology, Evolution, and Organismal Biology, Iowa State University, Iowa, Ames, United States of America; American Museum of Natural History, United States of America

## Abstract

**Background:**

Homoscleromorpha is the fourth major sponge lineage, recently recognized to be distinct from the Demospongiae. It contains <100 described species of exclusively marine sponges that have been traditionally subdivided into 7 genera based on morphological characters. Because some of the morphological features of the homoscleromorphs are shared with eumetazoans and are absent in other sponges, the phylogenetic position of the group has been investigated in several recent studies. However, the phylogenetic relationships within the group remain unexplored by modern methods.

**Methodology/Principal Findings:**

Here we describe the first molecular phylogeny of Homoscleromorpha based on nuclear (18S and 28S rDNA) and complete mitochondrial DNA sequence data that focuses on inter-generic relationships. Our results revealed two robust clades within this group, one containing the spiculate species (genera *Plakina*, *Plakortis*, *Plakinastrella* and *Corticium*) and the other containing aspiculate species (genera *Oscarella* and *Pseudocorticium*), thus rejecting a close relationship between *Pseudocorticium* and *Corticium*. Among the spiculate species, we found affinities between the *Plakortis* and *Plakinastrella* genera, and between the *Plakina* and *Corticium*. The validity of these clades is furthermore supported by specific morphological characters, notably the type of spicules. Furthermore, the monophyly of the *Corticium* genus is supported while the monophyly of *Plakina* is not.

**Conclusions/Significance:**

As the result of our study we propose to restore the pre-1995 subdivision of Homoscleromorpha into two families: Plakinidae Schulze, 1880 for spiculate species and Oscarellidae Lendenfeld, 1887 for aspiculate species that had been rejected after the description of the genus *Pseudocorticium*. We also note that the two families of homoscleromorphs exhibit evolutionary stable, but have drastically distinct mitochondrial genome organizations that differ in gene content and gene order.

## Introduction

Sponges (phylum Porifera) are exclusively aquatic and predominantly filter-feeding animals that play an important role in benthic ecosystems. There are currently 8,366 described species in the phylum (World Porifera Database), subdivided into three classes on the basis of body plan features: Calcarea Bowerbank, 1864 [1]; Demospongiae Sollas, 1885 [2] and Hexactinellida Schmidt, 1870 [3]. While recent phylogenetic studies have failed to find an obvious consensus concerning the monophyly *vs* paraphyly of Porifera and their exact branching relatively to other non-bilaterians (*i.e.* cnidarians, ctenophores, placozoans) [4], [5], [6], [7], [8], [9], it is now clear that Homoscleromorpha (Dendy, 1905) [10], previously defined as part of the Demospongiae, is the fourth high-level sponge taxon, alongside the three classically recognized classes (for recent references see [8], [11], [12]).

Homoscleromorphs are a small group (<100 described species) of exclusively marine sponges, generally located in shallow waters from 8 to 60 m, but also at more than 1000 m depth [13]. All species are dwellers of hard substratum communities often in semi-dark or dark conditions; some may grow only on coralligenous substratum. In some places, homoscleromorphs can be predominant and they seem to be strong competitors for space, overgrowing massive sponges, sea fans and erect bryozoans [13], [14], [15]. Their fossil record dates back at least to the Early Carboniferous [16], and is also documented in the Early and Upper Jurassic [17]. This fossil record, however, is poor, due to the homoscleromorph's reduced and poorly organized siliceous skeleton (when present), and provides no indication regarding their affinities and evolution.

**Figure 1:**
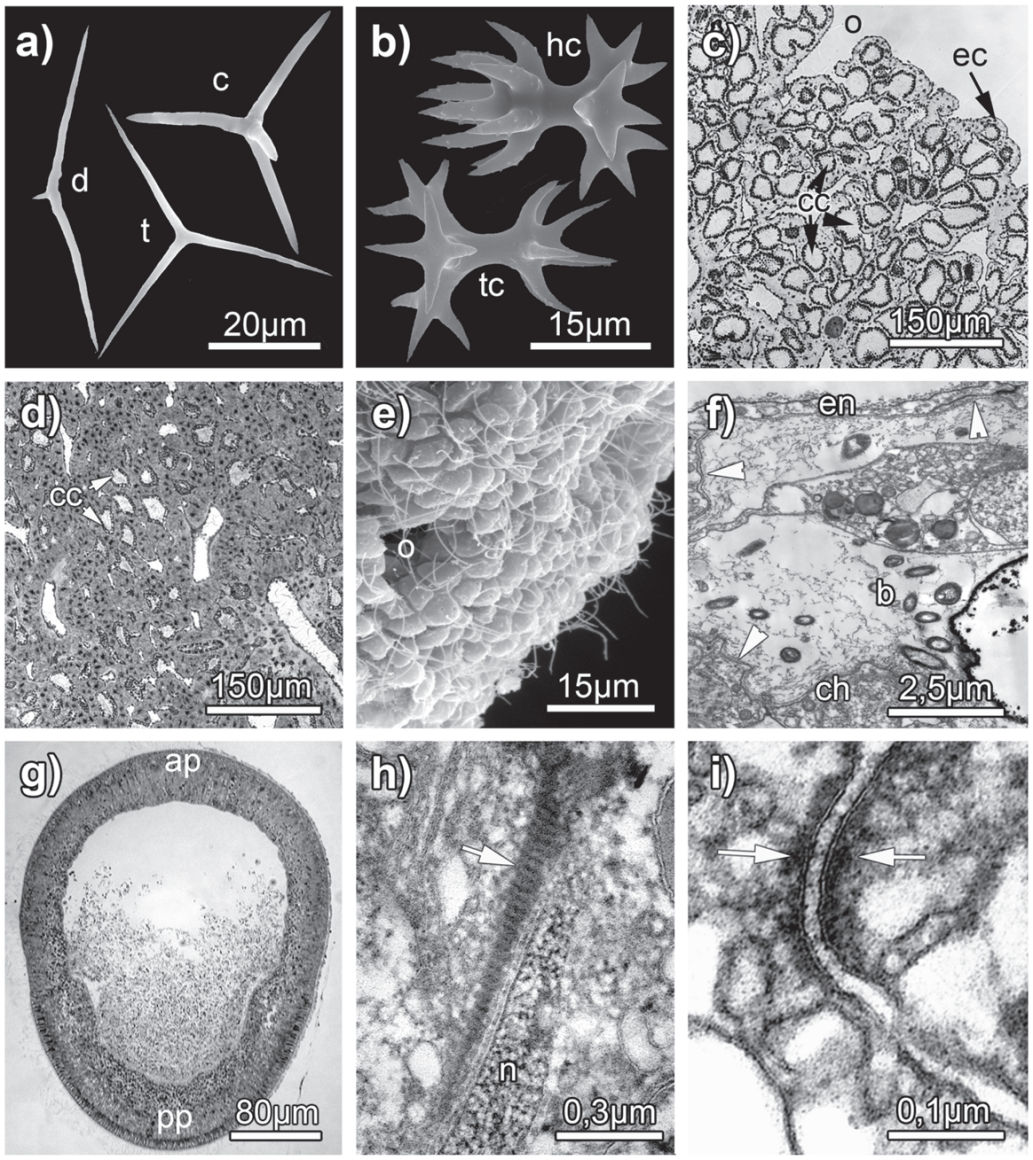
A few relevant morphological characters in the Homoscleromorpha. (a, b) spicules of the Homoscleromorpha (SEM). (a): *c* - calthrop of *Plakina trilopha* (Marseilles, Mediterranean); *d* – diod of *Plakina trilopha* (Marseilles, Mediterranean); *t* – triod of *Plakortis simplex* (Marseilles, Mediterranean); (b): *hc* – heterolophose calthrop (candelabra) of *Corticium candelabrum* (Adriatic Sea); *tc* – tetralophose calthrop of *Plakina weinbergi* (Mediterranean, Lebanon). (c) – *Oscarella kamchatkensis* (Avacha Bay, Bering Sea, Russia), transverse semi-thin section showing the sylleibid aquiferous system with eurypylous choanocyte chambers (*cc*), fine ectosome (*ec*) and an ostium (*o*); (d) – *Pseudocorticium jarrei* (Marseilles, Mediterranean) transverse semi-thin section showing the leuconoid aquiferous system with diplodal choanocyte chambers (*cc*). (e) – *Oscarella malakhovi* (Japan Sea, Russia) SEM micrograph of the flagellated exopinacoderm with ostium (*o*). (f) – *Oscarella viridis* (Marseilles, Mediterranean) TEM micrograph showing basement membrane (arrow heads) underlining the endopinacocytes (*en*) and choanocytes (*ch*) and endobiotic bacteria (*b*) in the mesohyl. (g) – *Corticium candelabrum* (Marseilles, Mediterranean) transverse semi-thin section of cinctoblastula larva, showing anterior (*ap*) and posterior (*pp*) poles. (h) – *Plakina trilopha* TEM micrograph of the cross-striated ciliar rootlet (arrow) close to a nucleus (*n*) in cinctoblastula larva. (i) – *Oscarella microlobata* (Marseilles, Mediterranean) TEM micrograph of cell junctions (*zonula adhaerens*) between the ciliated cells of cinctoblastula larva (arrows).

Although Homoscleromorpha show a great variability of forms, their general organization and the shared features of their cytology and embryology, as putative autapomorphic characters, argue for the monophyly of this group (Fig. 1). This sponge clade is characterized by an aquiferous system of either sylleibid-like or leuconoid organization with eurypylous, diplodal or aphodal choanocyte chambers (Fig. 1c, d). As far as skeletal structures are concerned, they harbor a peculiar type of tetractines spicules (calthrops), distinguishable from calthrops of the Demospongiae and their derivatives by their small size, ramification of one to all four actines (lophose calthrops) or reduction (diods and triods) (Fig. 1a, b) and by the presence of an amorphous axial filament [18], [19]. These spicules do not form a well-organized skeleton. Homoscleromorpha possess flagellated exopinacocytes and endopinacocytes (Fig. 1e), peculiar flagellated apopylar cells, a cinctoblastula larva (Fig. 1g), cross-striated ciliar rootlets in larval cells (Fig. 1h), a basement membrane underlying both choanoderm and pinacoderm (Fig. 1f), and *zonula adhaerens* cell junctions in adults and larval epithelia (Fig. 1i), as well as an asynchronous spermatogenesis (for review see [13], [15]). Some of these features are shared with Eumetazoa, making this group especially interesting.

Traditionally, Homoscleromorpha has been classified as a family or a suborder of the subclass Tetractinellida, within the class Demospongiae, mainly due to the shared presence of siliceous tetractinal-like calthrop spicules [20]. Lévi (1973) later proposed to classify them as a distinct subclass of the Demospongiae [21], a rank maintained in the most authoritative recent classification “Systema Porifera: a guide to the classification of sponges” [22]. However, the inclusion of Homoscleromorpha in the Demospongiae has been challenged by molecular studies [8], [9], [11], [23], [24]. Among these recent phylogenetic studies based on nuclear sequences, two alternative positions of homoscleromorphs have emerged: (i) closer to eumetazoan than to the other sponges, resulting in the paraphyly of sponges [7], [9], [11], [23], [24] or (ii) as the sister group of calcareous sponges within monophyletic Porifera [8], [12]. Recent studies based on complete mitochondrial genome sequences strongly supported the grouping of Homoscleromorpha with other sponges rather than with the Eumetazoa, although calcareous sponges were not included in the dataset [25]. Interestingly, a shared ancestry of Homoscleromorpha and Calcarea had been suggested earlier [26], [27], based on a slight similarity of spicule form and of larva type. However, these morphological characters were not convincing: indeed (i) the calthrop-like spicules are of a different mineralogical composition (calcium carbonate or silica); furthermore, (ii) the analogy between their larva types was based only on the use of a similar term for larvae that are now considered as clearly different, respectively amphiblastula for Calcarea and cinctoblastula for Homoscleromorpha [28].

Until 1995, two families were recognized within the Homoscleromorpha, Plakinidae Schulze, 1880 [29] and Oscarellidae Lendenfeld, 1887 [30], distinguished by the presence or absence of the mineral skeleton, respectively. However, in 1990, the discovery of a skeleton-less *Corticium*-like species led Solé-Cava et al. (1992) to propose the rejection of the family Oscarellidae [31]. Later, when this species was described as a member of a new genus *Pseudocorticium* [32], all homoscleromorph genera were merged into a single family, the Plakinidae. *Pseudocorticium* is indeed devoid of a mineral skeleton like the genus *Oscarella*, but is more similar in histological traits (notably the leuconoid aquiferous system and a well developed ectosome) to the spiculated genus *Corticium*. Thus, the Homoscleromorpha are currently considered to contain a single family, the Plakinidae Schulze, 1880 [29], including 7 genera (*Oscarella* Vosmaer, 1887 [33]; *Plakina* Schulze, 1880 [29]; *Plakortis* Schulze, 1880 [29]; *Plakinastrella* Schulze, 1880 [29]; *Corticium* Schmidt 1862 [34]; *Pseudocorticium* Boury-Esnault et al., 1995 [32]; *Placinolopha* Topsent, 1897 [35]) and about 78 species. The genera are distinguished mainly by four morphological characters [13], [14], [32]: the presence or absence of a siliceous skeleton; the presence or absence of a cortex associated with the architecture of the aquiferous system and type of choanocyte chambers; if spicules are present, the number of spicule size classes and the presence and type of ramification in the actins of calthrops.

Several recent molecular phylogenetic studies examined internal relationships within three of the four major sponge groups: Calcarea [36], [37], Demospongiae, [23], [38], [39] and Hexactinellida [12]. Because the Homoscleromorpha were only recently recognized as a separate, major sponge clade [7], [8], [9], [11], [12], [23], [24], their internal relationships have not yet been investigated using molecular markers. However, such investigation is necessary, given both the potential usefulness of Homoscleromorpha in pharmacological research [40] and the recent emergence of two *Oscarella* species (*O. lobularis* and *O. carmela*) as models in the Evo-Devo field [15], [41], [42], [43]. Here we present a thorough investigation of homoscleromorph phylogeny using morphology, 18S rDNA, 28S rDNA and complete mitochondrial genome sequence data. Our aims were to test the two competing hypotheses for their broadest subdivision proposed in the literature, to test genus validity and to formulate hypotheses concerning morphological character evolution within the group.

## Methods

### Specimen Collection

Specimens from the Mediterranean Sea and the North Pacific were collected by SCUBA diving or provided to us by colleagues (see Acknowledgments). Locations of the collection sites are shown on a geographical map (Fig. S1). The species used in this study, their current taxonomic status, and their NCBI GenBank sequence accession numbers are summarized in Table 1. The identification of all specimens used in this study has been carefully checked on the basis of morphological characters by the taxonomists in our team.

**Table 1:** List of species used in this work according to the classification of Systema Porifera [22] and the recent update undertaken in the World Porifera Database [90].

				GenBank accession numbers			Collection sites
				18S rRNA	28S rRNA	Mitochondrial genome	
Plakinidae	N°						
		***Oscarella***	**Vosmaer, 1877**				
	1	*Oscarella lobularis*	(Schmidt, 1862)	**HM118536**	**HM118549**	**HQ269361**	Marseilles, France (Coral cave or Plane Island)
	2	*Oscarella carmela*	Muricy & Pearce, 2004	EU702422	EF654519	NC_009090	California, USA (Carmel Bay)
	3	*Oscarella malakhovi*	Ereskovsky, 2006	**HM118537**	**HM118550**	**HQ269364**	Japan Sea, Russia (Vostok Bay, )
	4	*Oscarella microlobata*	Muricy et al., 1996a	**HM118538**	**HM118551**	**HQ269355**	Marseilles, France (Jarre Cave)
	5	*Oscarella tuberculata*	(Schmidt, 1868)	**—**	**—**	**HQ269353**	Marseilles, France (Coral Cave)
	6	*Oscarella viridis*	Muricy et al., 1996a	**—**	**—**	**HQ269358**	Marseilles, France (Jarre Cave)
		***Pseudocorticium***	**Boury-Esnault et al., 1995**				
	7	*Pseudocorticium jarrei*	Boury-Esnault et al., 1995	**HM118539**	**HM118552**	**HQ269357**	Marseilles, France (Jarre Cave)
		***Corticium***	**Schmidt, 1862**				
	8	*Corticium candelabrum*	Schmidt, 1862	**HM118540**	**HM118553**	**HQ269363**	Marseilles, France (Coral Cave)
	9	*Corticium* sp.1	n/a	**HM118541**	**HM118554**	—	Palau (Ngedesakr Channel)
	10	*Corticium* sp.2	n/a	**HM118542**	**HM118555**	—	Vanuatu
		***Plakortis***	**Schulze, 1880**				
	11	*Plakortis simplex*	Schulze, 1880	AY348884	**HM118556**	**HQ269362**	La Ciotat, France (3 PP Cave)
	12	*Plakortis halichondrioides*	(Wilson, 1902)	**HM118543**	**HM118557**	**HQ269359**	Bocas del Toro, Panama
		***Plakina***	**Schulze, 1880**				
	13	*Plakina jani*	Muricy et al., 1998	**HM118544**	**HM118558**	**HQ269360**	Marseilles or La Ciotat, France (Jarre Cave, 3 PP Cave)
	14	*Plakina crypta*	Muricy et al. 1998	**HM118545**	**HM118559**	**HQ269352**	La Ciotat, France (3 PP Cave)
	15	*Plakina trilopha*	Schulze, 1880	**HM118546**	**HM118560**	**HQ269356**	Marseilles, France (Jarre Cave)
	16	*Plakina monolopha*	Schulze, 1880	**HM118547**	**HM118561**	**HQ269351**	Sète, France (Thau pond)
	17	*Plakina* sp.	n/a	**—**	**—**	**HQ269354**	Marseilles, France (Plane island)
		***Plakinastrella***	**Schulze, 1880**				
	18	*Plakinastrella onkodes*	(Uliczka, 1929)	**HM118548**	**HM118562**	—	Panama (Bocas del Toro)
	19	*Plakinastrella* sp. *	n/a	EU702423	—	EU237487	Florida, USA (Looe Keys)
	20	*Plakinastrella* sp. 2 *3269*	n/a	—	**HM118563**	—	Coral Sea Queensland, Australia (Holmes Reef)

The collection sites and the GenBank numbers of the 18S and 28S rDNA sequences and of the complete mitochondrial genomes are indicated. In the sequences column, the new sequence accession numbers are mentioned in bold. A number is given to each species for the understanding of the map in Fig. S1. Note: an asterisk (*) indicates that this species has been previously misidentified as *Plakortis angulospiculatus* and published under this name. n/a: not available.

### DNA Sequence Acquisition

#### Nuclear markers: 18S and 28S rRNA genes

Procedures used for genomic DNA extraction, cloning and DNA sequencing are described in previous studies [44], [45], except for a few DNA extractions made with the QIAamp DNA mini kit (DNA purification from tissues, Qiagen), following the manufacturer's instructions. PCR primers for full-length/partial 18S and partial 28S ribosomal DNA (rDNA) amplification are provided in Table S1a. Reaction mixes were adapted from [44], [45]. Most 18S and 28S rDNA amplicons were obtained by nested PCR with different combinations of primers. Thermocycling was often carried out using the “touchdown” protocols with annealing temperatures ranging from 65°C to 45°C, (depending on the primer melting temperature) and a range of cycles from 30 to 45 were performed. As protocols had to be adapted for each species, the exact conditions of amplification are not listed here but can be obtained from the authors upon request.

Despite substantial efforts (variety of DNA extraction procedures tested, PCR additive tested, gradient PCR tested), we were not able to obtain 18S/28S sequences for some species (especially a *Placinolopha* species), and in some cases only shorter sequences could be amplified (see Table 1 for distribution of missing data). This may be due to either poor conservation of samples or to PCR-inhibitors (pigments or secondary metabolites for example).

#### Complete mitochondrial genome

The overall procedure for complete mtDNA sequencing was described in [46]. For this study, a partial cytochrome *b* sequence was determined for all collected specimens and used to design Homoscleromorpha-specific primers. In addition, demosponge-optimized primers for large and small subunit rRNA, and, if necessary, species-specific primers for other genes, were used to amplify the complete mtDNA for each species in 2–4 fragments (Table S1b). PCR reactions for each species were combined in equimolar concentration, sheared and barcoded as described in [47]. Barcoded PCR fragments were combined together and used for the GS FLX Titanium library preparation (454 Life Sciences). Pyrosequencing was carried out on a Genome Sequencer FLX Instrument (454 Life Sciences) at the University of Indiana Center for Genomics and Bioinformatics. The STADEN package v. 1.6.0 (http://staden.sourceforge.net) was used to assemble the sequences. Gaps and uncertainties in the assembly were filled/resolved by primer-walking using conventional Sanger sequencing. tRNA genes were identified with the tRNAscan-SE program [48]; other genes were identified by similarity searches in local databases using the FASTA program [49] and in GenBank using BLAST (http://blast.ncbi.nlm.nih.gov). All new sequences were deposited in GenBank under accession numbers listed in Table 1.

### Sequence Alignment and Phylogenetic Analysis

#### Nuclear loci

To achieve a reasonable trade-off between representativeness of outgroup taxa and ease of alignment, and because our prime interests were relationships within the Homoscleromorpha, we restricted our sampling to sponges and included only a few members of two key sponge groups as outgroup: Calcarea and Demospongiae (sequences from GenBank). Thus, one Calcinea, two Calcaronea and two Halichondrida were added to our Homoscleromorpha sampling. Initial sequence alignment was performed using the software MUSCLE available online (http://www.ebi.ac.uk/Tools/muscle/index.html) [50], [51], and subsequently optimized by eye using the Bioedit Sequence Alignment Editor v5.09 [52]. Ambiguously aligned regions were determined by the program Gblocks v0.91 b [53]. A relaxed selection of blocks is better for short alignment [54], thus the settings were the following for the 18S rDNA [1: 12; 2: 17; 3: 4; 4: 3; 5: all] and 28S rDNA [1: 11; 2: 11; 3: 4; 4: 4; 5: all]. The treatment by GBlock resulted in the removal of 4%, and 9% for the 18S and 28S alignments respectively. The character exclusion sets based on Gblocks are available upon request from the corresponding author. Phylogenetic analyses were performed using parsimony, maximum likelihood (ML) and Bayesian methods.

For maximum parsimony (MP) analyses, we used MEGA v4.0 [55]. Characters were always treated as unordered and equally weighted. We performed heuristic searches with 100 replicates of random taxon addition sequence and TBR branch swapping. For ML analyses we used JModelTest [56] to determine the best-fitting nucleotide substitution model for each data set. This resulted, according to the likelihood score, [Akaike Information Criterion (AIC)], in the following model choice for respectively 18S and 28S: TIM2+Γ+I (TIM2 model+gamma (Γ)-distributed rates of substitution among sites+estimated proportion of invariant sites) and GTR+Γ (general time-reversible substitution model+gamma (Γ)-distributed rates of substitution among sites [57], [58]), (with the following parameters, for 18S: I = 0.4290, α = 0.5200; for 28S: α = 0.4280); Then, we performed the analyses with the PhyML software v3 [59], [60] using the previously estimated parameters. Among sites rate heterogeneity was estimated using a discrete approximation of the gamma distribution with 4 rate categories. For both methods, gaps were treated as missing data and the statistical robustness of the tree topology was assessed by non-parametric bootstrap resampling (1000 replicates) [61].

In molecules constrained by secondary structure such as 18S and 28S rRNA, the nucleotides involved in stems and loops do not evolve independently, as assumed with standard models of substitution [62], [63] such as those compared in Modeltest [64] and jModelTest [56]. Mixed models of substitution, in which a matrix describes the changes among nucleotide pairs and another matrix is fitted for single nucleotide changes, thus potentially provide a better fit to nucleotide sequence data from such molecules [12], [37], [45], [65]. We thus conducted Bayesian analyses with partitioning of our datasets in stems and loops. The pre-requirement of this method is to determine a consensual secondary structure from several sequences. For this purpose, we used the online RNAalifold software (http://mobyle.pasteur.fr/cgi-bin/portal.py, with the MFE (minimum free energy) fold algorithm) that calculates consensus secondary structures for a set of aligned RNAs [66]. Then, we used the PHASE software (http://www.bioinf.manchester.ac.uk/resources/phase) providing such partitioned models and performing Bayesian phylogenetic inferences [67]. We chose the RNA7D model for nucleotide pairs, also known as OTRNA [68], in which seven pair states are considered (AU, UA, GC, CG, GU, UG and MM for all mismatches). This model is a biologically plausible restriction of the most general model involving those seven pairs, considering transition, double transition, and transversion rates. It uses seven frequency parameters and four rate parameters (the most common seven state model has 21 rates). For unpaired nucleotides, we used the REV model. For both models, we used a gamma distribution with six categories to account for rate variation among sites. The program *mcmcphase* used Markov chain Monte Carlo (MCMC) to sample from the posterior probability distribution of phylogenetic trees, branch lengths and sequence evolution model parameters. For the burn-in, the number of burn-generations was set at 150,000 generations. The posterior distributions (thinning) were calculated sampling every 150 generations. The total run length was 1,500,000 generations. For priors and other MCMC parameters, we used default values proposed by the program. We checked for convergence by examining the likelihood values (PLT file), and ran four chains for each analysis with different random seeds five times to check whether the same stationary distribution was reached. The program *mcmcsummarize* then provided the topology, branch lengths and branch support of the consensus tree. Bayesian posterior probabilities (PP) were used for assessing the confidence value of each node. Lacking positions were scored as missing data.

The 18S and 28S alignments plus the resulting trees (in ML) have been deposited on the free TreeBASE database (numbers 10402 and 10403 for the 18S and 28S Matrix ID respectively) (http://www.treebase.org, [69], [70]). Given that bootstrap proportion values (BP values) are a conservative measure of a clade support [71] and that Bayesian posterior probabilities (PP values) might overestimate node support [72], PP values >95% and BP values >85% were interpreted as giving significant support to the respective clades.

#### Mitochondrial coding sequences

Mitochondrial coding sequences for *Cantharellus cibarius* and *Capsaspora owczarzaki* mtDNA were downloaded from http://megasun.bch.umontreal.ca/People/lang/FMGP/proteins.html. Other sequences were derived from the GenBank files: *Acanella eburnea* NC_011016, *Acropora tenuis* NC_003522, *Astrangia* sp. NC_008161, *Briareum asbestinum* NC_008073, *Chrysopathes formosa* NC_008411, *Metridium senile* NC_000933, *Nematostella* sp. NC_008164, *Pocillopora damicornis* NC_009797, *Porites porites* NC_008166, *Ricordea florida* NC_008159, *Sarcophyton glaucum* AF064823, AF063191, *Savalia savaglia* NC_008827, *Aurelia aurita* NC_008446, *Amphimedon compressa* NC_010201, *Aplysina fulva* NC_010203, *Ephydatia muelleri* NC_010202, *Halisarca dujardini* NC_010212, *Igernella notabilis* NC_010216, *Oscarella carmela* EF081250, *Plakinastrella* sp. NC_010217, *Tethya actinia* NC_006991, *Xestospongia muta* NC_010211, *Trichoplax adhaerens* NC_008151, Placozoan BZ10101 NC_008832, *Amoebidium parasiticum* AF538042–AF538052, *Monosiga brevicollis* NC_004309, *Allomyces macrogynus* NC_001715, *Mortierella verticillata* NC_006838, *Rhizopus oryzae* NC_006836.

Amino acid sequences of individual proteins were aligned three times with ClustalW v1.82 [73] using different combinations of opening/extension gap penalties: 10/0.2 (default), 12/4 and 5/1. The three alignments were compared using SOAP [74], and only positions that were aligned identically among them were included in phylogenetic analyses. Nucleotide sequences of protein-coding genes were aligned based upon amino-acid alignments using CodonAlign [75].

We assembled two datasets of concatenated mitochondrial amino-acid sequences. The first, “small”, dataset (3662 amino acid positions) encompassed 35 taxa representing Homoscleromorpha, Demospongiae, and Anthozoa. The second, “large”, dataset included sequences from additional outgroup taxa: 4 species of fungi, the ichthyosporean *Amoebidium parasiticum*, the amoeba *Capsaspora owczarzaki*, the choanoflagellate *Monosiga brevicollis*, two placozoans, and the scyphozoan *Aurelia aurita* (45 taxa, 3278 amino acids). The differences in size among these datasets are due to the fact that there are more positions than can be reliably aligned when fewer taxa are included. Furthermore, we constructed a concatenated alignment of nucleotide sequences (10,986 bp) for Cnidaria and Demospongiae corresponding to the small amino-acid dataset.

Phylogenetic analyses on the nucleotide data were conducted with the RAxML v7.0.4 [76] using the GTR+Γ model of sequence evolution with 4 discrete categories of gamma-distributed among-site rates. Phylogenetic analyses on concatenated amino-acid datasets were carried out with RAxML and PhyloBayes (PB) v3.2d [77], [78]. For the RAxML analyses on concatenated data, we used ProtTest v2.4 [79] to evaluate different models of amino-acid substitution. With the same combination of Γ and F (F stands for empirical amino-acid frequencies) parameters, JTT [80] was preferred among standard matrices for the small dataset, while cpREV [81] was the best-fit model for the larger dataset according to the likelihood score, Akaike Information Criterion (AIC = −2lnL+2K, where L is the likelihood and K is the number of parameters) and Bayesian Information Criterion (BIC = −2lnL+Klogn, where n is the number of variable characters). We used the category CAT+Γ [72] as well as CAT+GTR+Γ models for the PhyloBayes analysis and ran four chains until convergence (max diff <0.1), sampling every 10^th^ tree.

All alignments and resulting trees have been deposited on TreeBASE (number 10864 Matrix ID, http://www.treebase.org; [69], [70]).

#### Statistical tests of alternative topologies

When topologies were not totally congruent between markers or phylogenetic methods, we tested alternative topologies for each dataset. ML analyses for alternative topologies were conducted in PAUP* v4.0b10 (for rRNA data [75]) and PAML v4.4b (for mtDNA data [76]). Alternative topologies were compared with the approximately unbiased (AU) [82] and weighted Shimodaira and Hasegawa tests (WSH) [83] using CONSEL (with default values for RELL calculation: 10 sets of bootstrap replicates; each set consists of 10000 replicates) [84]. Results are summarized in Table 2.

**Table 2:** Statistical tests results of alternative topologies in maximum likelihood framework.

Data sets	Topologies constrained	*P* AU Test	*P* WSH Test
18S rDNA	Monophyly of genus *Oscarella*	0.191	0.244
	Monophyly of genus *Plakortis*	0.324	0.328
	Monophyly of the genus *Plakina*	0.014*	0.044*
28S rDNA	Paraphyly of genus *Oscarella*	0.018*	0.033*
	Paraphyly of genus *Plakortis*	0.008*	0.023*
	Monophyly of the genus *Plakina*	0.138	0.173
Mitochondrial genome	Monophyly of genus *Oscarella*	0.112	0.187
	B2 non valid	0.364	0.554
	Polyphyly of *Plakina*+B2 non valid	0.495	0.662

Asterisk (*) indicates a significant *P* value (*P*<0.05), and thus the rejection of the hypothesis mentioned. AU = Approximately Unbiased, WSH = weighted Shimodaira–Hasegawa. Graphic representations of topologies constrained are available upon request to the corresponding author.

## Results

### Nuclear markers 18S and 28S rRNA genes

**Figure 2:**
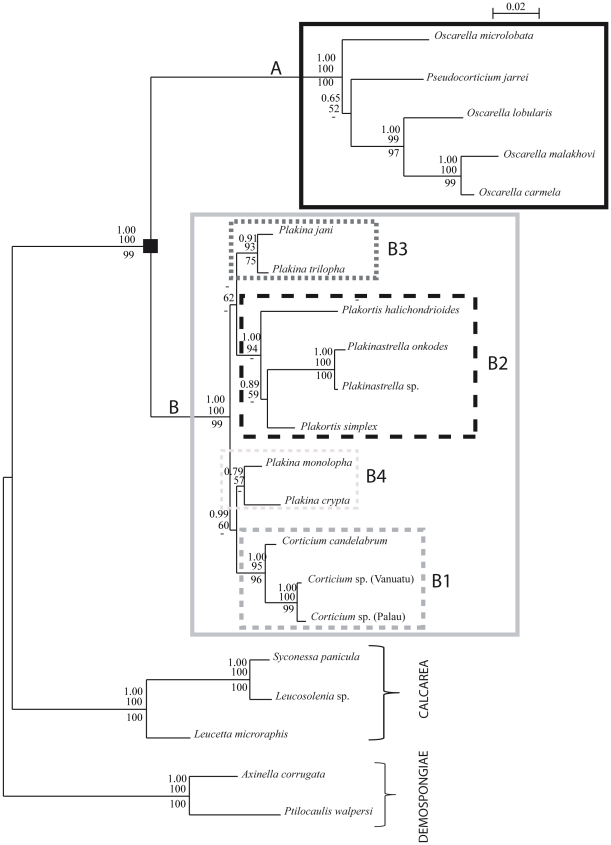
Phylogram showing the relationships among the six genera of Homoscleromorpha based on 18S rDNA analyses. The topology presented corresponds to the ML analysis. Outgroups are Calcarea (AM180965, AM180976, AF100945) and Demospongiae (AY737637, AY737638) species. The Homoscleromorpha species are split into two robust clades: A and B. The numbers are from top to bottom: posterior probabilities for BI and bootstrap values (>50) for ML and MP respectively. Bayesian and MP analyses recovered slightly different phylogenies (Fig. S2). The black square points out the node corresponding to Homoscleromorpha.

**Figure 3:**
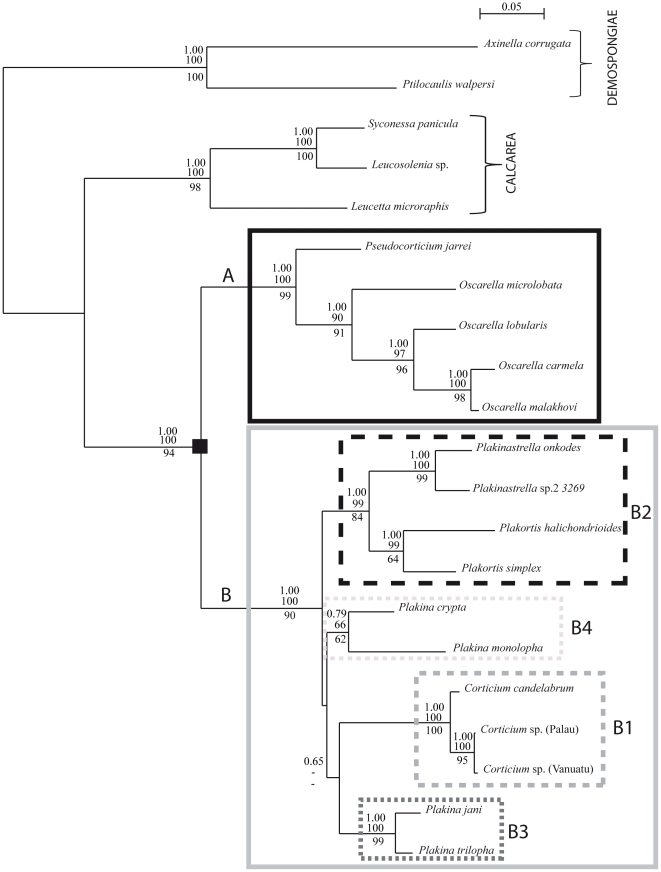
Phylogram showing the relationships among the six genera of Homoscleromorpha based on 28S rDNA analyses. The topology presented corresponds to the ML analysis. Outgroups are Calcarea (AM180995, AM181007, AY026372) and Demospongiae (AY864741, AY864743) species. The Homoscleromorpha species are split into two robust clades: A and B. The numbers are from top to bottom: posterior probabilities for BI and bootstrap values (>50) for ML and MP respectively. Bayesian and MP analyses recovered slightly different phylogenies (Fig. S3). The black square points out the node corresponding to Homoscleromorpha.

The results obtained by the 18S and 28S using the different phylogenetic methods were mostly congruent. We chose to present the topologies obtained by ML method for each marker, indicating for each node the support found by the different methods (Figs. 2 and 3). All the other trees obtained by the different methods are provided in figures S2 and S3.

Homoscleromorpha are divided into two clades supported by high BP and PP values (from 90 to 100 for BP and PP = 1.00): one groups *Pseudocorticium* and *Oscarella* genera (clade A) while the other groups *Corticium*, *Plakina*, *Plakinastrella* and *Plakortis* genera (clade B).

Inside clade A, the monophyly of the genus *Oscarella* depends on the fluctuating positions of *O. microlobata* and *Pseudocorticium jarrei*: *Oscarella* is monophyletic according to the 28S topologies *vs* paraphyletic according to the 18S topologies. The longer branch leading to *O. microlobata* suggests that these unstable positions may be due to long-branch attraction (LBA) artifact. Statistical tests (AU and WSH) indicate that we cannot reject the hypothesis of the monophyly of *Oscarella* with the 18S dataset, while the hypothesis of a monophyletic clade containing *Oscarella* species+*Pseudocorticium jarrei*, with *O. microlobata* at the base of the tree, can be rejected according to the 28S dataset (Table 2).

Among the three other analyzed species of *Oscarella*, *O. malakhovi* and *O. carmela* (both from North Pacific) are closer to each other (maximum BP and PP in all analyses) than they are to the Mediterranean species *O. lobularis*.

Inside clade B, the three species of *Corticium* form a highly supported monophyletic group (clade B1 supported by robust values from 95 to 100 for BP and PP = 1.00), where the two south Pacific species are sister groups. The four species belonging to *Plakortis* and *Plakinastrella* group together in most analyses, forming a robust clade B2 (BP from 84 to 99 and maximum PP). In this clade B2, the two *Plakinastrella* species are grouped (maximum PP and BP from 99 to 100) while the relative position of the two species of *Plakortis* is uncertain. These relationships are thus congruent with the monophyly of *Plakinastrella* whereas the monophyly of *Plakortis* would have to be further tested. Nevertheless, statistical tests indicate that we cannot reject significantly the hypothesis of the monophyly of *Plakortis* with the 18S dataset, while the hypothesis of the paraphyly of *Plakortis* can be rejected with the 28S dataset (Table 2). As far as genus *Plakina* is concerned (all species from the north-west of the Mediterranean Sea), it does not appear to be monophyletic: on the one hand *P. trilopha* and *P. jani* form a supported group (clade B3 supported by PP values ranging from 0.91 to 1.00 and BP values from 75 to 100), on the other hand *P. monolopha* and *P. crypta* have a weaker affinity to one another (clade B4 not supported). B3 and B4 do not group together whatever the marker or the phylogenetic method used. Nevertheless, regarding the low support values supporting the relationships between B1, B2, B3 and B4, we chose to statistically test the hypothesis of monophyly of the genus *Plakina* (Table 2). In contrast to the 28S rDNA dataset, we can reject significantly the monophyly of *Plakina* with the 18S rDNA dataset.

### Mitochondrial genome evolution in Homoscleromorpha

**Figure 4:**
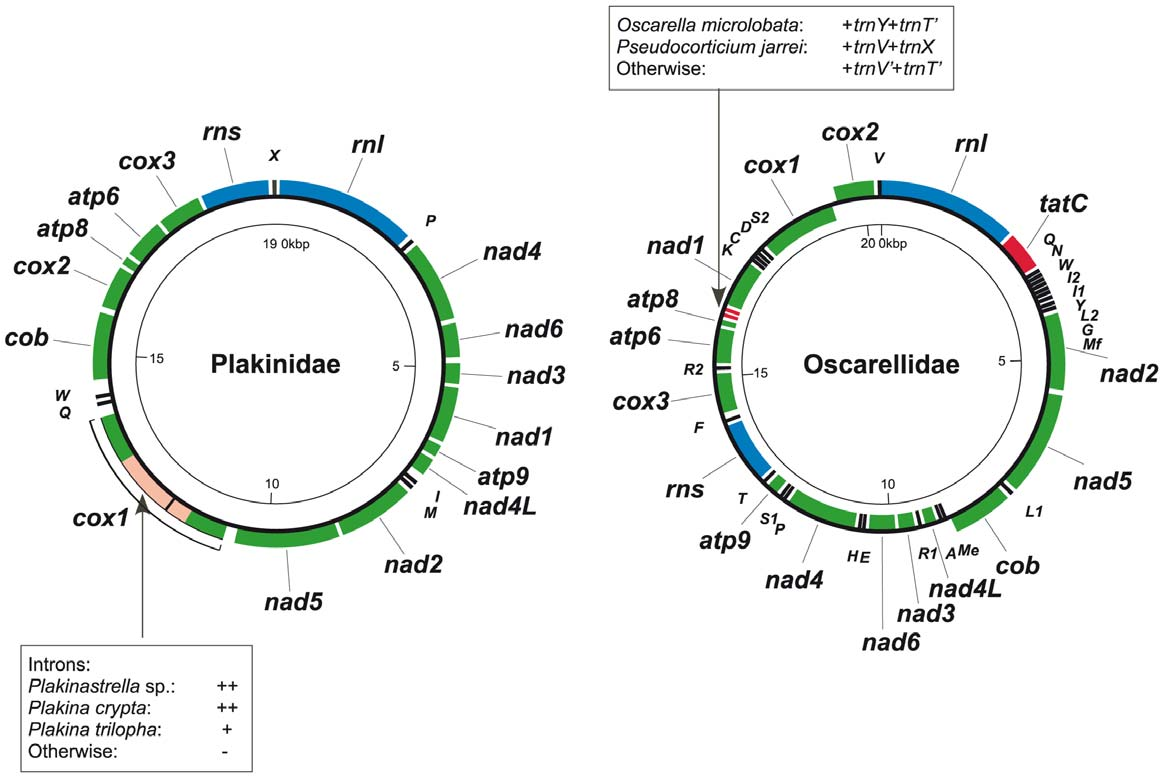
Mitochondrial genome organization in Plakinidae and Oscarellidae. Protein (green) and ribosomal (blue) genes are *atp6*, *atp8–9*: subunits 6, 8, and 9 of F0 adenosine triphosphatase (ATP) synthase; *cob*: apocytochrome b; *cox1–3*: cytochrome c oxidase subunits 1–3; *nad1–6* and *nad4L*: NADH dehydrogenase subunits 1–6 and 4L; *rns* and *rnl*: small and large subunit rRNAs; *tatC*: twin-arginine translocase component C. tRNA genes (black) are identified by the one-letter code for their corresponding amino acid. Genes outside the main circle are transcribed clock-wise, inside – counter clock-wise. Variations within each genome organization are shown in red and explained in corresponding boxes.

We determined the complete mitochondrial genome sequences of 12 species of homoscleromorphs representing five genera in this group plus the partial sequences of two species (*Oscarella malakhovi* and *Plakina trilopha*). These mitochondrial genomes can be subdivided into two groups on the basis of their mitochondrial genome organization (Fig. 4). All genomes in the first group (*Oscarella* and *Pseudocorticium* species) are very similar to the mitochondrial genome of *Oscarella carmela* [85] and share with it a nearly identical gene order, the presence of *tatC*, a gene for subunit C of the twin arginine translocase, as well as genes for 27 tRNAs [85]. The genomes in the second group (*Plakina*, *Plakinastrella*, *Plakortis* and *Corticium* species) have a genome organization that is very similar to that in *Plakinastrella* sp. (previously misidentified as *Plakortis angulospiculatus*) [25], displaying a nearly identical gene arrangement, lack of *tatC* as well as 20 of the 25 tRNA genes typically found in demosponges. These two different genome organizations have a clearly defined phylogenetic distribution within the Homoscleromorpha: all spiculate homoscleromorphs have *Plakinastrella* sp.-like mitochondrial genome organization, while all aspiculate homoscleromorphs have *O. carmela*-like mitochondrial genome organization. The variations within each group are minor. Within the *Oscarella*-like genomes, two duplicated tRNA genes (V and T) have changed identities in some species. Within the *Plakinastrella*-like genomes, one or two introns are present in *cox1* of *Plakinastrella* sp., *Plakina crypta* and *Plakina trilopha* but absent elsewhere, an observation we interpret as multiple independent losses [86].

### Mitochondrial genome phylogenetic analyses

**Figure 5:**
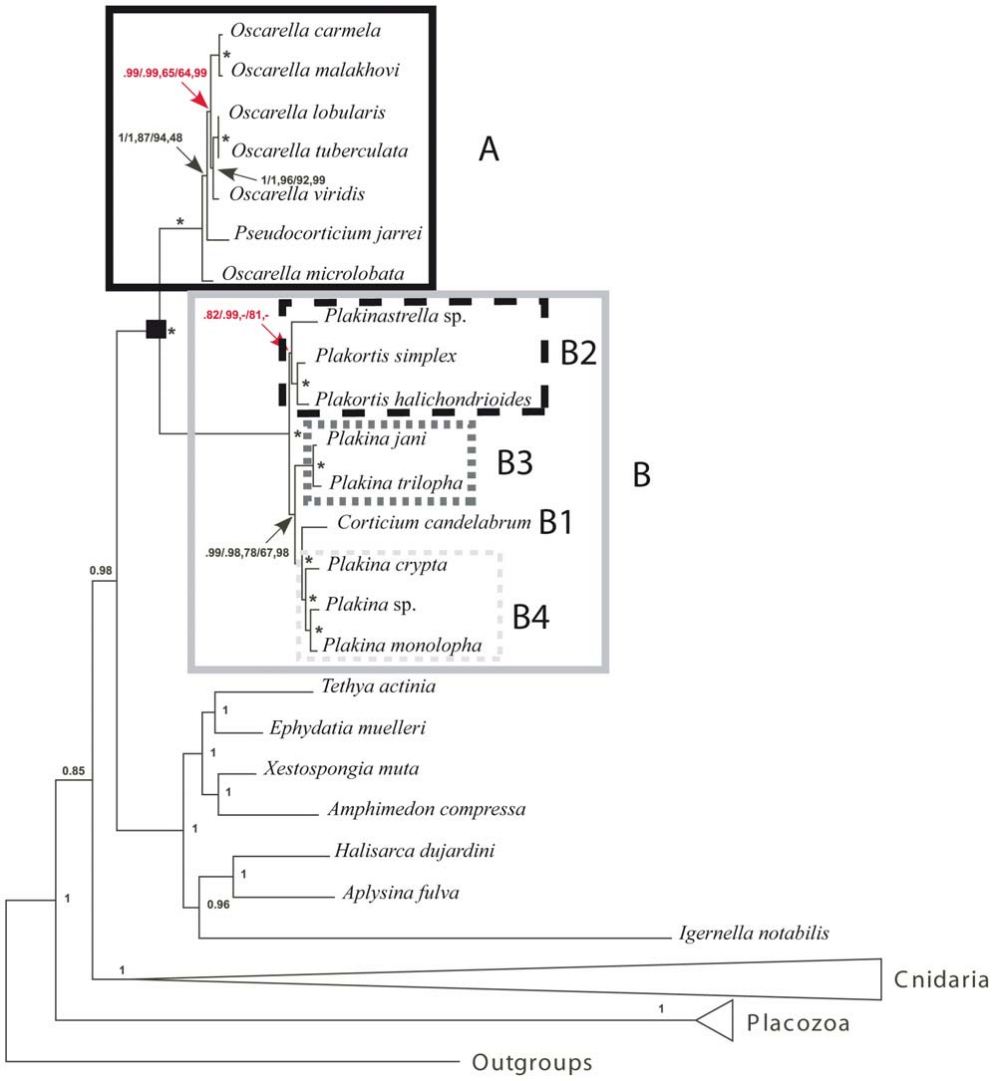
Homoscleromorph relationships based on the analyses of concatenated sequences from 14 mitochondrial protein genes. Bayesian tree obtained from the analysis of 3278 aligned amino acid positions for the 45 taxa with the CAT+GTR model is shown. Identical relationships within Homoscleromorpha were inferred using Bayesian analyses with the CAT+GTR model and Maximum Likelihood analyses of the small (35 taxa) amino acid dataset. Bayesian analyses with the CAT model as well as ML analysis of the nucleotide dataset and of the 45 taxa amino acid dataset resulted in slightly different phylogenies (Fig. S4). Asterisks indicate nodes within Homoscleromorpha with maximum support values in all analyses. For other nodes within this group, support values represent (from left to right): posterior probabilities in Bayesian analysis using CAT+GTR model with 45/35 taxa, bootstrap support values for the ML analyses of amino-acid datasets with 45/35 taxa, and bootstrap support values for the ML analysis of the 35 taxa nucleotide dataset. Two unstable nodes are shown in red with a minus sign indicating that the node was not recovered in the analysis. For nodes outside Homoscleromorpha, only posterior probability values for the Bayesian analysis with the CAT+GTR model and 45 taxa are shown.

Mitochondrial coding sequences were previously shown to be highly informative for reconstructing phylogenetic relationships among non-bilaterian animals [38]. Here we used them to conduct Bayesian and ML phylogenetic analyses of homoscleromorph relationships, on two datasets that included sequences from 16 species of homoscleromorphs but differed by the number and diversity of outgroups (see Material and Methods). The results of these analyses were highly congruent (Fig. 5 and Fig. S4). All homoscleromorphs are subdivided into two groups corresponding to aspiculate species (genera *Oscarella* and *Pseudocorticium*, clade A) and spiculate species (genera *Plakina*, *Plakortis*, *Plakinastrella*, and *Corticium*, clade B). Among aspiculate species *Oscarella microlobata* always forms the sister taxon to a clade grouping other *Oscarella* species and *Pseudocorticium*, rendering *Oscarella* paraphyletic. Other *Oscarella* species form two groups that reflect their geographical location: one composed of Mediterranean species *O. lobularis*, *O. tuberculata*, and *O. viridis* and another – of Pacific species *O. carmela* and *O. malakhovi*. The phylogenetic position of *Pseudocorticium jarrei* switches between being (i) the sister group to all *Oscarella* except *O. microlobata* (all ML analyses; BP analyses under the CAT+Γ+GTR model) and (ii) being the sister group to the Pacific species of *Oscarella* (BP analyses under the CAT+Γ model). Among spiculate species three clades are well supported in all analyses: one containing two *Plakortis* species, the second grouping *Plakina trilopha* and *P. jani*, and the third including the remaining three *Plakina* species plus *Corticium candelabrum* as their sister taxon. The latter two clades (all *Plakina* species+*C. candelabrum*) were always placed together in phylogenetic analyses based on mitochondrial data, but with variable statistical support. The phylogenetic position of *Plakinastrella* sp. differs between analyses. It forms the sister group to all other spiculate species in RAxML analysis based on the large dataset of amino acid sequences and based on the small dataset of nucleotide sequences, but groups with the two *Plakortis* species in all other analyses (Fig. S4). Despite strong statistical support for several clades in our phylogenetic analyses of mitochondrial data, some alternative topologies (especially within the clade B) cannot be rejected in ML topology tests (Table 2). For example, the monophyly of the genus *Oscarella* is only rejected with 89 probability, while alternative positions of *Plakinastrella* are practically undistinguishable from this perspective. Finally, our analysis shows that there is relatively little mitochondrial genetic diversity within either spiculate or aspiculate homoscleromorphs, although the divergence between the two groups is substantial.

## Discussion

### The position of *Pseudocorticium* and the restoration of two families within the Homoscleromorpha

In 1995, Boury-Esnault et al. described a new genus of Homoscleromorpha, *Pseudocorticium* [32]. The name for this genus was chosen on the basis of its morphological similarity to *Corticium*, in particular the presence of the cortex. However, unlike *Corticium*, *Pseudocorticium* does not produce silicious spicules. It has therefore been suggested that *Pseudocorticium* may represent an aspiculate morph of *Corticium*, unable to secrete spicules in an environment poor in silica, a case that has been reported for some demosponges [87]. The grouping of *Pseudocorticium* with *Corticium* received some support from an allozyme analysis where *Pseudocorticium jarrei* (identified as *Corticium* sp. or *Corticium*-like in the cited paper) was found to be more closely related to *Corticium candelabrum* than to *Oscarella lobularis* and *O. tuberculata* [31].

**Figure 6:**
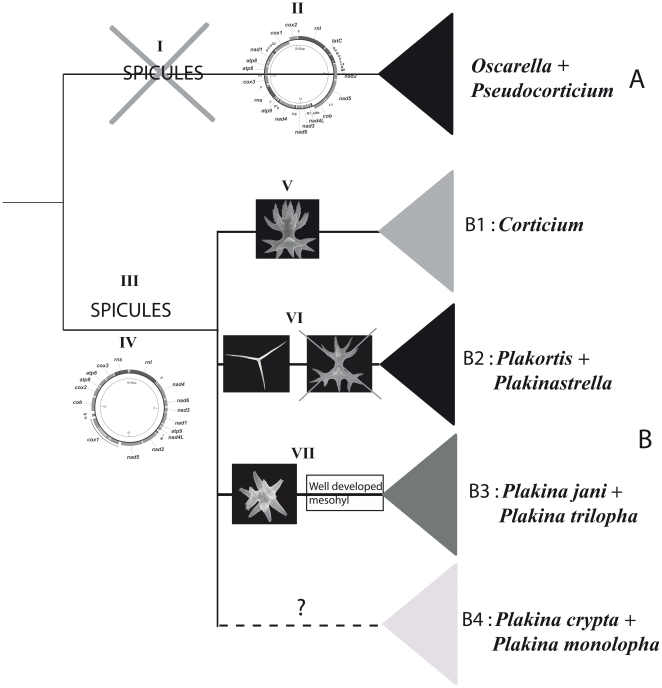
Simplified consensus tree showing the genera relationships in Homoscleromorpha based on molecular phylogenies. Morphological characters that are diagnostics of these clades are mapped. I: Absence of spicules in clade A. II: Specific gene arrangement in mitochondrial genome in clade A (see Fig. 4). III: Presence of spicules (lophate and alophate) in clade B. IV: Specific gene arrangement in mitochondrial genome in clade B (see Fig. 4). V: Presence of a candelabra (heterolophose calthrop) specific to *Corticium* genus, clade B1. VI: Presence of alophose spicules and absence of lophose spicules in clade B2. VII: Presence of tetralophose calthrop and of a well developed mesohyl in clade B3.

By contrast, our phylogenetic analyses, based on nuclear and mitochondrial markers, as well as the overall mitochondrial genome organization, reject the hypothesis of close relationship between *Pseudocorticium* and *Corticium* and, instead, place *Pseudocorticium* with the genus *Oscarella*. This result leads to the subdivision of the Homoscleromorpha into two clades (A and B): one comprising only aspiculate species (clade A: *Pseudocorticium* and *Oscarella*), the other grouping spiculate species (clade B: *Plakina*, *Plakortis*, *Plakinastrella* and *Corticium*). Our results are thus congruent with the subdivision of homoscleromorphs into two families, Oscarellidae Lendenfeld, 1887 [30] (corresponding to clade A minus the *Pseudocorticium* genus that was described latter) and Plakinidae Schulze, 1880 [29] (corresponding to clade B), as was accepted prior to 1995 on the basis of absence/presence of a mineral skeleton (Fig. 6).

From a morpho-anatomical point of view, the separation of spiculate and aspiculate homoscleromorphs into two clades, makes the secondary loss of spicules in Oscarellidae or the gain of spicules in Plakinidae equally parsimonious reconstructions. Our results also indicate that the similar cortex, aquiferous system organization and outer morphological similarities encountered between *Corticium* and *Pseudocorticium* represent either homoplasic or plesiomorphic characters. As a consequence of our phylogenetic analyses, we propose to restore the family Oscarellidae Lendenfeld, 1887 [30] with the following diagnosis: “Homoscleromorpha without skeleton, with a variable degree of ectosome development. The aquiferous system has a sylleibid-like or leuconoid organization, with eurypylous or diplodal choanocyte chambers.” The diagnosis for the family Plakinidae should be modified from that proposed by Schulze, 1880 [29] as follows: “Homoscleromorpha with a skeleton formed by a combination of small calthrops and/or derivatives through reduction (diods and triods), generally arranged uniformly in the sponge body, surrounding the aquiferous system in a regular “alveolar” way or more confusedly dispersed. The aquiferous system has a sylleibid-like or leuconoid organization, with eurypylous or aphodal choanocyte chambers”. Accordingly, the definitions of the two families are:

Family Plakinidae: “Homoscleromorpha with inorganic spicular skeletal complements, represented by calthrops, diods and triods; with a sylleibid-like or leuconoid aquiferous system made up of eurypylous or aphodal choanocyte chambers”.

Family Oscarellidae: “Homoscleromorpha without spicules, with a sylleibid-like or leuconoid aquiferous system made up of spherical, eurypylous or diplodal choanocyte chambers”.

On the basis of the above phylogenetic revision we propose new taxonomical keys for Homoscleromorpha families:

**Key to families** (modified from [13])

With inorganic (spicular) skeletal complement**Plakinidae**

Lacking inorganic skeletal complement**Oscarellidae**

### The genus *Oscarella*, monophyletic or paraphyletic?

Phylogenetic analyses based on three different markers used in this study produced conflicting results with regard to the monophyly *vs.* paraphyly of *Oscarella*. The analyses of mtDNA and 18S rRNA data support the paraphyly of *Oscarella*, *O. microlobata* either grouping with *Pseudocorticium jarrei* (18S) or forming a sister group to all other aspiculate homoscleromorphs (mtDNA, highly supported). In contrast to that, the analyses of 28S rRNA sequences produce a monophyletic *Oscarella* genus with *Pseudocorticium jarrei* as its sister group. Despite these uncertainties, statistical tests do not allow us to fully reject the monophyly of *Oscarella*. As morphological characters are not helpful here, a more detailed molecular study comprising more numerous *Oscarella* species and more molecular loci is needed to resolve this issue.

### A possible common origin of *Plakinastrella* and *Plakortis* genera

Most of our analyses also tentatively support the grouping of *Plakortis* and *Plakinastrella*. While the affinity between these two genera has never been previously proposed, it is worth noticing that this clade is in fact supported by a morphological synapomorphy. Indeed, in both genera, lophose spicules are absent, in contrast to all the other spiculate genera, which possess at least one type of lophose spicules (Fig. 6). The two genera remain, nevertheless, distinct. *Plakortis* has diods and triods of a single size class, whereas, *Plakinastrella* synthesizes diods, triods and/or calthrops of several size classes [13]. In all our analyses based on nuclear rDNA data, the three species of *Plakinastrella* form a monophyletic group, thus making the combination of those spicules a valid morphological character to define this genus. Even if weakly supported in rRNA analyses, the monophyly of *Plakortis* cannot be rejected and is strongly supported by mitochondrial data and spicular characters. As in the case of *Oscarella*, a molecular phylogenetic analysis encompassing more *Plakortis* species will be necessary to investigate these relationships.

### The challenging of the *Plakina* genus: need for substantial nomenclature revision

Genus *Plakina* has been defined as “Plakinidae with a spiculation of diods, triods and calthrops in a single size class, and with homolophose calthrops with, one, two, three, or four lophate rays” [13]. The presence of such lophose calthrops and the lack of the heterolophose calthrop (“candelabra”) distinguish *Plakina* from *Corticium*, with which it shares some spicule similarity. However, the boundaries between the two genera remain unstable, primarily due to the scarcity of informative morphological characters, and several species originally assigned to *Corticium* were transferred to *Plakina* [88]. Interestingly, a close relationship between the *Corticium* genus (clade B1) and some *Plakina* species (clade B4) was found in molecular analyses with the 18S rDNA marker as well as with mitochondrial genomes. Moreover, the four studied *Plakina* species plus *Corticium* form a monophyletic group in mitochondrial genome analyses. Several authors have previously recognized that *Plakina* is probably a paraphyletic genus [13], [88], [89], a supposition strongly supported by our study. Indeed, *Plakina* species were never recovered as a monophyletic group in our analyses (regardless of genetic marker and analytical method). Non-monophyly of *Plakina* may explain the wide variability in morphological characters previously observed in this genus [88]. Thus, the genus *Plakina* should be redefined and, potentially subdivided into several genera on the basis of a comprehensive analysis of extant species. One of the new genera might contain *Plakina jani* and *P. trilopha* (clade B3, always recovered in all of our analyses) that are characterized by the presence of a well developed mesohyl, well-differentiated ectosome, large subectosomal cavities and a tetralophose calthrop (Fig. 6). All these characters are absent in the other *Plakina* (*P. monolopha* and *P. crypta*) included in our study.

### The monophyly of *Corticium* genus: valid morphological characters

The three species of *Corticium* used for the nuclear rRNA analysis form a well-supported monophyletic group. Thus, the spicular characteristics that are used as diagnosis of this genus “a spiculation consisting almost exclusively of non-lophose calthrops in one size class and heterolophose calthrops (candelabra)” appear to be valid [13]. Among those characters, the presence of candelabra, a special kind of tetralophose calthrops (Fig. 1b) is the best morphological apomorphy of this genus.

In conclusion, this study represents the first attempt to elucidate with molecular tools the phylogeny of the Homoscleromorpha, a small group of sponges that has been recently recognized as the fourth major lineage in the phylum Porifera, using both nuclear and mitochondrial molecular markers as well as morphological characters. As the result of our study we propose to restore the pre-1995 subdivision of the Homoscleromorpha into two families: Plakinidae Schulze, 1880 for spiculate species and Oscarellidae Lendenfeld, 1887 for aspiculate species that had been abandoned after the description of the genus *Pseudocorticium*. These two families are well supported in all our phylogenetic analyses and display evolutionary stable, but clearly distinct mitochondrial genome organizations that differ in gene content and gene order.

Our results also reject the monophyly of the genus *Plakina*, and question the monophyly of *Oscarella*, necessitating further studies of these genera. In fact, a more detailed study of *Pseudocorticium* and *Oscarella* species is currently in progress in our laboratories. Furthermore, the monophyly of *Plakortis* should be tested using more comprehensive taxon sampling and the phylogenetic position of *Placinolopha*, which was not included in our molecular analyses, should be investigated.

Finally, this study illustrates once again that the combination of several molecular markers is a powerful tool for the *a posteriori* re-examination of morphological characters and the reassessment of those that can or cannot be used as diagnostic features for the definition of taxa. This study contributes to the improvement of our knowledge of the metazoan Tree of Life – a highly necessary endeavour for both ecological and evolutionary studies.

## Supporting Information

Figure S1 Locations of the collections sites
(7.29 MB TIF)

Figure S2 Trees resulting from the MP and Bayesian analyses with the 18S rDNA marker. The numbers correspond to posterior probabilities for BI and bootstrap values MP analyses.
(1.28 MB TIF)

Figure S3 Trees resulting from the MP and Bayesian analyses with the 28S rDNA marker. The numbers correspond to posterior probabilities for BI and bootstrap values MP analyses.
(1.28 MB TIF)

Figure S4 Additional trees resulting from the ML and Bayesian analyses in mitochondrial (small and large datasets).
(2.47 MB TIF)

Table S1 a) List of primers names and sequences used in this study, for 18S, 28S rDNA and complete mitochondrial genome amplifications. b) Primer pairs used for mtDNA amplification and number of long PCR realized for each species.
(0.08 MB DOC)
