# Supplementary figures and images for: Molecular Phylogeny Restores the Supra-Generic Subdivision of Homoscleromorph Sponges (Porifera, Homoscleromorpha)
Source: PLoS One. 2010 Dec 14;5(12):e14290. doi: 10.1371/journal.pone.0014290 (PMC3001884; doi:10.1371/journal.pone.0014290)

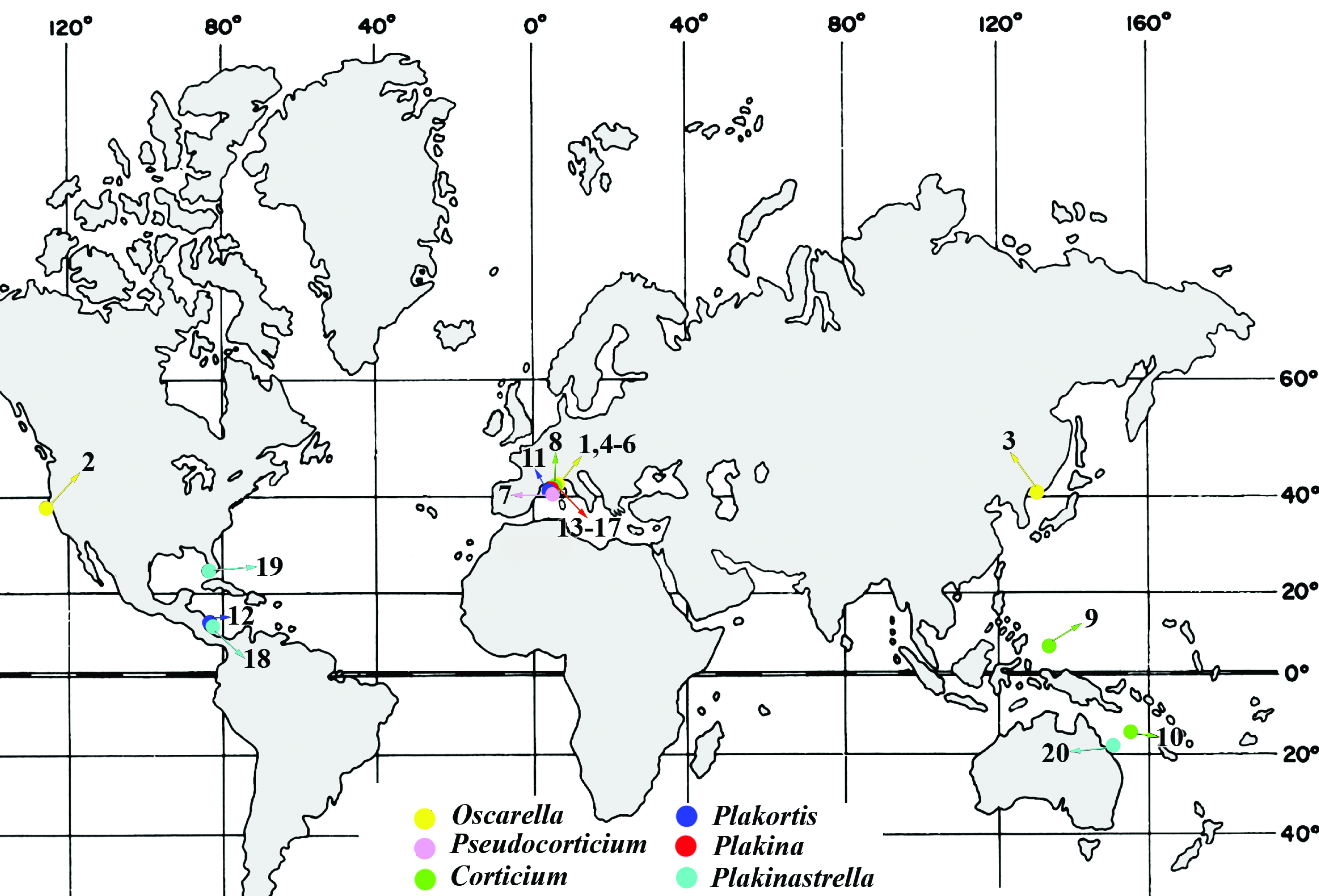

Supplement: Figure S1 — Locations of the collections sites (7.29 MB TIF) [file pone.0014290.s001.tif]

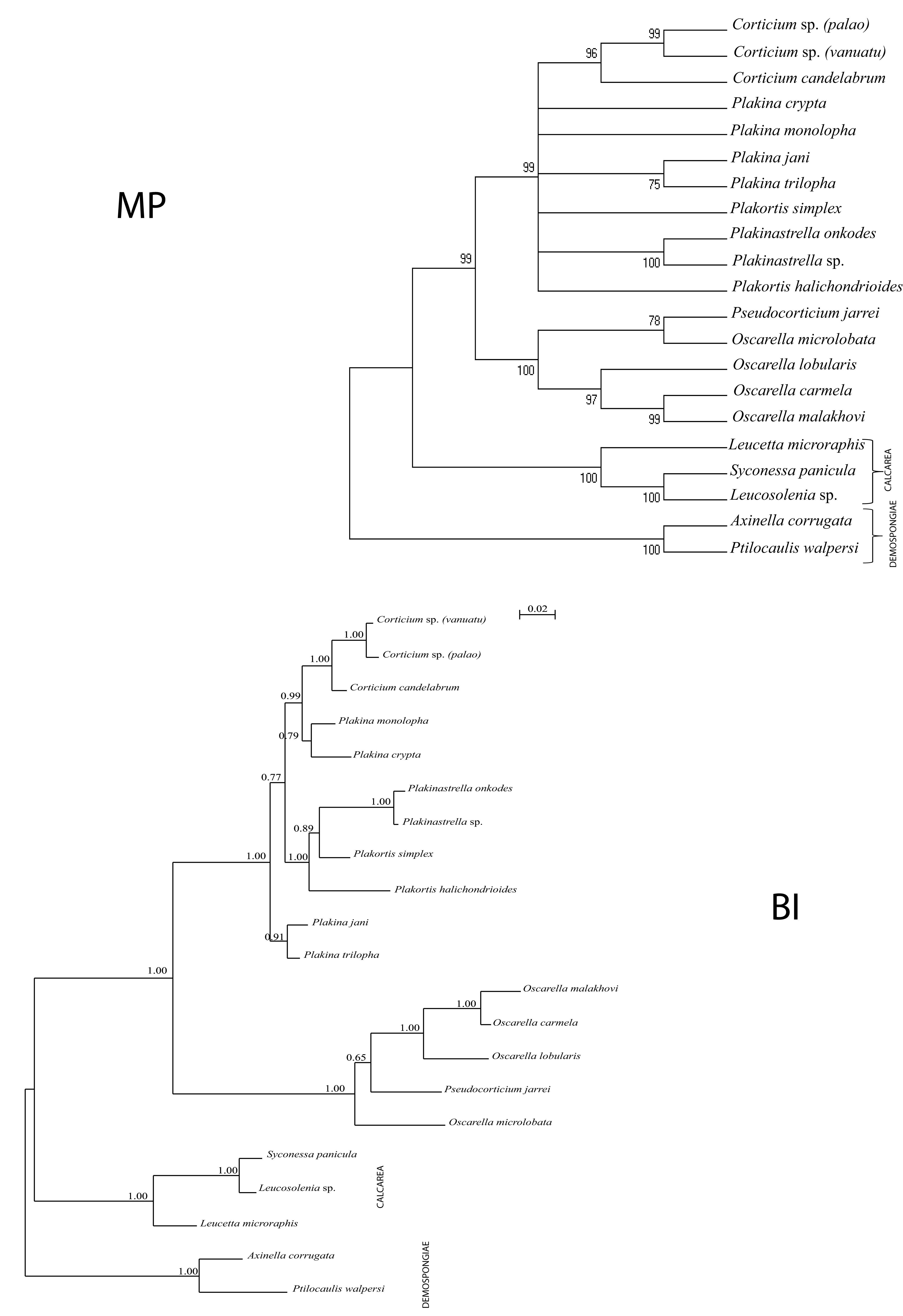

Supplement: Figure S2 — Trees resulting from the MP and Bayesian analyses with the 18S rDNA marker. The numbers correspond to posterior probabilities for BI and bootstrap values MP analyses. (1.28 MB TIF) [file pone.0014290.s002.tif]

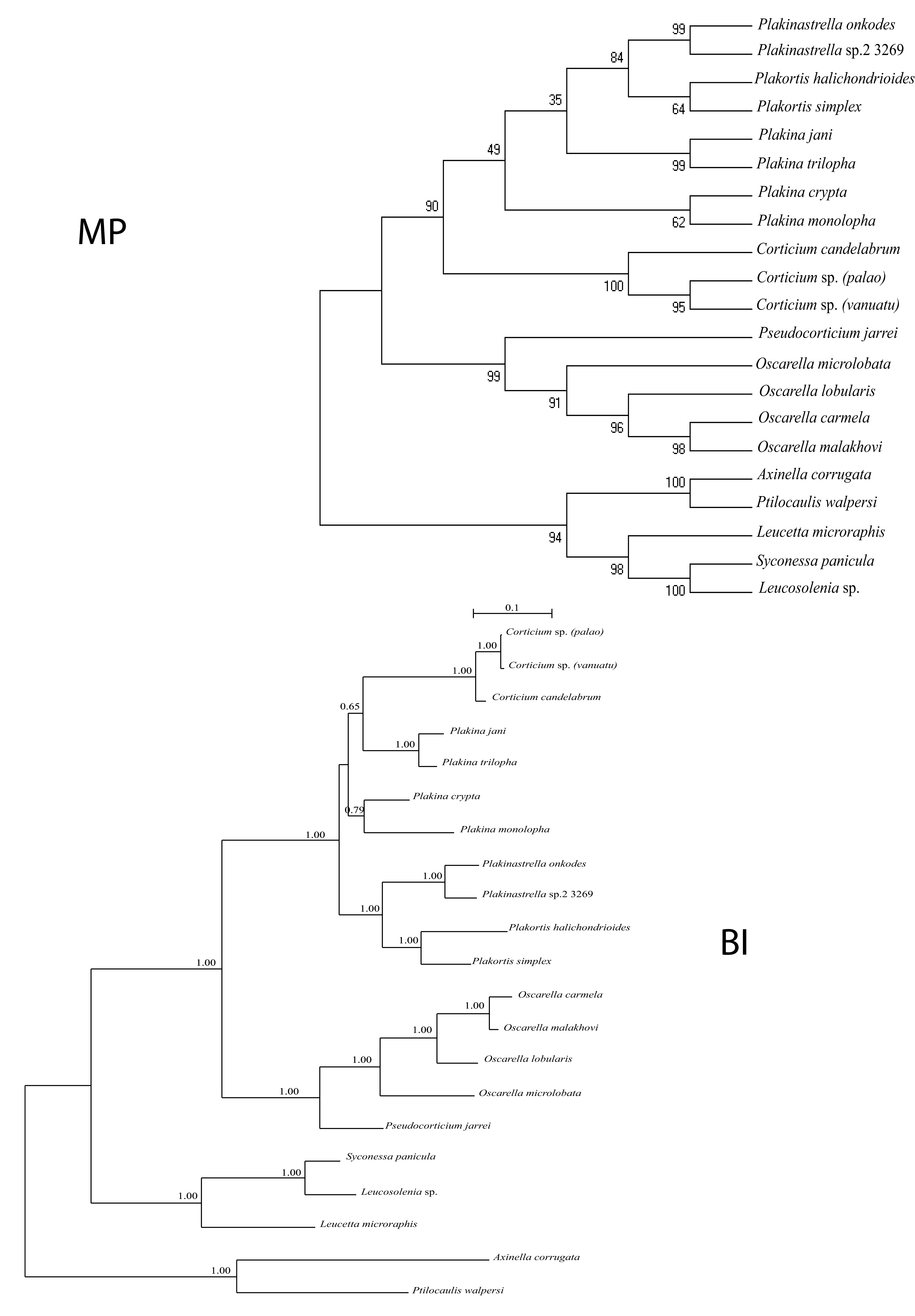

Supplement: Figure S3 — Trees resulting from the MP and Bayesian analyses with the 28S rDNA marker. The numbers correspond to posterior probabilities for BI and bootstrap values MP analyses. (1.28 MB TIF) [file pone.0014290.s003.tif]

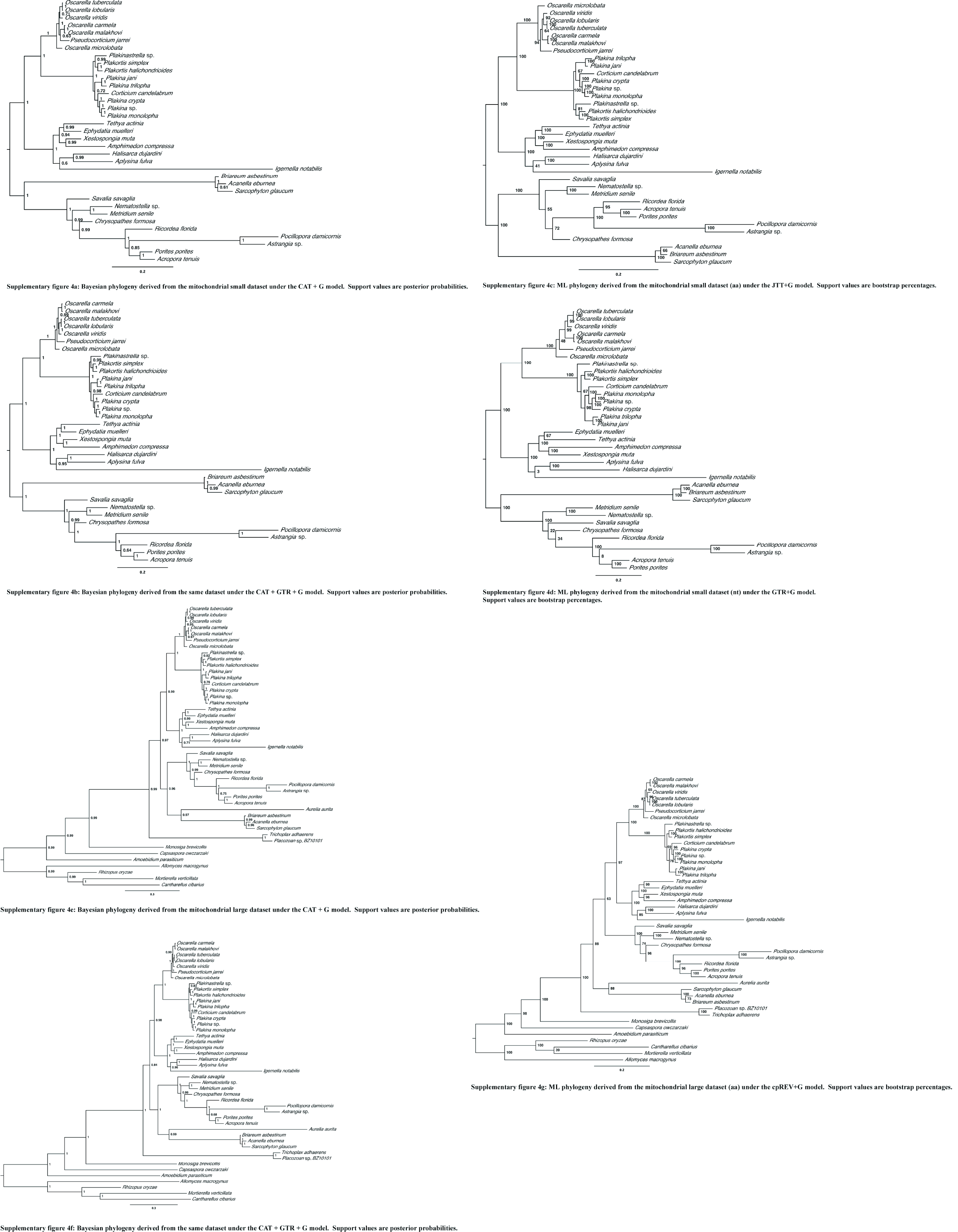

Supplement: Figure S4 — Additional trees resulting from the ML and Bayesian analyses in mitochondrial (small and large datasets). (2.47 MB TIF) [file pone.0014290.s004.tif]
