# Supplementary material for: Molecular Phylogeny Restores the Supra-Generic Subdivision of Homoscleromorph Sponges (Porifera, Homoscleromorpha)
Source: PLoS One. 2010 Dec 14;5(12):e14290. doi: 10.1371/journal.pone.0014290 (PMC3001884; doi:10.1371/journal.pone.0014290)
Supplement: Table S1 — a) List of primers names and sequences used in this study, for 18S, 28S rDNA and complete mitochondrial genome amplifications. b) Primer pairs used for mtDNA amplification and number of long PCR realized for each species. (0.08 MB DOC) [file pone.0014290.s005.doc]

| **Gene** | **Primer name** | **Sequence (5’-3’)** | **References** |
| --- | --- | --- | --- |
| **rDNA** |  |  |  |
| 18S | (F) S1 | AAC CTG GTT GAT CCT GCC A | Borchiellini et al., 2001 |
|  | (R) S2 | TGC AGG TTC ACC TAC AGA A | Borchiellini et al., 2001 |
|  | (F) D | ACT GTG AAA CTG CGA ATG GCT C | This study |
|  | (R) G | CAC CTA CGG AAA CCT TGT TAC GCA | This study |
|  | 18SHomo F | GYG AAA CTG CGA ATG GCY C | This study |
|  | 18SHomo R | CTT GTT ACG ACT TTT ACY TCC TC | This study |
| 28S | (F) X | GAA AAG AAC TTT GRA RAG AGA GT | This study |
|  | (R) S2 | ATK CGY TTC CCT CCY AAC GG | Borchiellini et al., 2004 |
|  | (F) S1 | AGT CTT TCG CCC CTA TAC CCA | This study |
|  | (R) Y | ACC CGC TGA ATT TAA GCA T | Borchiellini et al., 2004 |
|  | 28SHomo F1 | CAT ATC AAT AAG CGG AGG AA | This study |
|  | 28SHomoF2 | GAG TCG GGT TGT TTG GGA | This study |
|  | 28SHomoR | ATC GAT TTG CAC GTC AGA | This study |
| **mtDNA** |  |  |  |
| *cob* | homo_p-cob-f1 | TTT TGR GCN GCA ACT GTT ATT AC | Conserved, this study |
|  | homo_p-cob-r1 | GGA TCN CCA AAC AAA TTR GGA GC | Conserved, this study |
|  | homo_o-cob-f1 | GGN GGN TAT AGT GTR TCT AAT GC | Conserved, this study |
|  | homo_o-cob-r1 | AAA TAC CAY TCT GGT CGT ATG TG | Conserved, this study |
|  | plakina-cob-r1 | GAT TCC AAT TGG ATT ATG AGA CC | Conserved, this study |
|  | Plakortis-cob-r1 | TTA CTA CCT ATA TAA GGG ATT GC | Conserved, this study |
|  | dl49-cob-f1 | AGA TTT GTT TGG TGC AAT GAT TC | Specific, this study |
| *cox1* | dl46-cox1-f1 | GGT TTG GTA ATT GAT TAT TAC CTC | Specific, this study |
|  | dl46-cox1-f1 | CCA GTA CCT GCT CCT TGT TCT AC | Specific, this study |
| *cox3* | dl49-cox3-f1 | CGG GAT GTT ATT AGA GAA TCG AC | Specific, this study |
|  | dl49-cox3-r1m | GTT TTA CTA TTT GAG TAT GGT GC | Specific, this study |
| *nad1* | dl30-nad1-f1 | ACG GAA AGT TTT AGG GTA TAT GC | Specific, this study |
|  | dl30-nad1-r1 | TTA CAC CAT CTG CTA TAG GTT GC | Specific, this study |
|  | dl49-nad1-f1 | TGT GTG GGC TCT CTA AAT GTG AC | Specific, this study |
|  | dl49-nad1-r1 | AAA ACA TTA TAG CAG CAG GGT AC | Specific, this study |
| *nad2* | dl24-nad2-f1 | ACT AAT CAT TGA GTT TAG CGG TC | Specific, this study |
|  | dl24-nad2-r1 | AAT TGC CAT AAT TGG GTG CAT CC | Specific, this study |
|  | dl48-nad2-f1 | GGT TCG TTA GAA AGT ATA CAA GC | Specific, this study |
|  | dl48-nad2-r1 | CCG CTA AAC TCA ATG ATT AGT GG | Specific, this study |
|  | dl49-nad2-f1 | AGT CCC TAA AGT TGT AAT ATT TGC | Specific, this study |
|  | dl49-nad2-r1 | ACC AAC GAT AAT ATC GCA CTT GC | Specific, this study |
| *nad5* | cb1-nad5-f1 | TAG TGT GAT GAA CGA GGT TAG TC | Specific, this study |
|  | cb1-nad5-r1 | GAA TGA CTA ACC TCG TTC ATC AC | Specific, this study |
|  | dl34-nad5-f1 | GAT TAG TAT TGG CGA TGT TTG TC | Specific, this study |
|  | dl34-nad5-r1 | CTC ATT CAT CAC ACT AAA TAC TG | Specific, this study |
| *rnl* | homo-rnl-f1 | AAA TTG AAT TCG TAG TGA AGA TGC | Conserved, this study |
|  | homo-rnl-r1 | ACC TTC GTT ACT TTT TAG AAG GC | Conserved, this study |
|  | diplo-rnl-f1 | TCG ACT GTT TAC CAA AAA CAT AGC | Lavrov et al., 2008 |
|  | diplo-rnl-r1 | AAT TCA ACA TCG AGG TSG GAA AC | Lavrov et al., 2008 |
| *rns* | demo-rns-f1 | GCA AAC AGG ATT AGA TAC CCT GG | Conserved, this study |
|  | demo-rns-r1 | GCG ATT TGT ACG AAC ACT AGA GC | Conserved, this study |

**Supplementary Table 1a: Primer names and sequences used for rDNA and mitochondrial amplifications.**

| **Species name (14)** | **# of long PCR amps** | **Primer combinations** |
| --- | --- | --- |
| *Oscarella lobularis* | 4 | homo-o-cob-r1+homo-rnl-f1; homo-o-cob-f1+demo-rns-f1; demo-rns-f1+dl46-cox1-f1; dl46-cox1-r1+homo-rnl-r1 |
| *Oscarella tuberculata* | 3 | homo-rnl-f1+homo-o-cob-r1; homo-o-cob-f1+demo-rns-f1; demo-rns-r1+homo-rnl-r1 |
| *Oscarella viridis* | 4 | homo-rnl-f1+homo-o-cob-r1; homo-o-cob-f1+demo-rns-f1; demo-rns-r1+dl30-nad1-f1; dl30-nad1-r1+homo-rnl-r1 |
| *Oscarella malakhovi* | 3 | homo-rnl-f1+homo-o-cob-r1; homo-o-cob-f1+demo-rns-f1; demo-rns-r1+diplo-rnl-r1 |
| *Oscarella microlobata* | 3 | homo-rnl-f1+homo-o-cob-r1; homo-o-cob-f1+demo-rns-f1; demo-rns-r1+homo-rnl-r1 |
| *Pseudocorticium jarrei* | 3 | homo-rnl-f1+homo-o-cob-r1; homo-o-cob-f1+demo-rns-f1; demo-rns-r1+homo-rnl-r1 |
| *Plakina monolopha* | 3 | homo-p-cob-f1+diplo-rnl-r1; diplo-rnl-f1+cb1-nad5-f1; plakina-cob-r1+cb1-nad5-f1 |
| *Plakina* sp. | 2 | homo-p-cob-f1+diplo-rnl-r1; homo-rnl-f1-plakina-cob-r1 |
| *Plakina crypta* | 2 | homo-p-cob-f1+diplo-rnl-r1; homo-rnl-f1-plakina-cob-r1 |
| *Plakina jani* | 2 | homo-p-cob-f1+diplo-rnl-r1; diplo-rnl-f1-plakina-cob-r1 |
| *Plakina trilopha* | 3 | homo-p-cob-f1+diplo-rnl-r1; homo-rnl-f1-dl24-nad2-r1; dl24-nad2-f1-plakina-cob-r1 |
| *Plakortis halichondrioides* | 3 | homo-p-cob-f1+diplo-rnl-r1; homo-rnl-f1-dl34-nad5-r1; dl34-nad5-f1-plakortis-cob-r1 |
| *Plakortis simplex* | 3 | homo-p-cob-f1+diplo-rnl-r1; homo-p-cob-r1+dl48-nad2-f1; diplo-rnl-f1+dl48-nad2-r1 |
| *Corticium candelabrum* | 5 | dl49-cox3-f1+diplo-rnl-r1; diplo-rnl-f1+dl49-nad1-r1; dl49-nad1-f1+dl49-nad2-r1; dl49-nad2-f1+homo-p-cob-r1; dl49-cob-f1-dl49-cox3-r1m |

**Supplementary Table 1b: Primer pairs used for mtDNA amplification**
